# DRI-MVSNet: A depth residual inference network for multi-view stereo images

**DOI:** 10.1371/journal.pone.0264721

**Published:** 2022-03-23

**Authors:** Ying Li, Wenyue Li, Zhijie Zhao, JiaHao Fan

**Affiliations:** 1 College of Computer Science and Technology, Jilin University, Changchun, China; 2 Key Laboratory of Symbolic Computation and Knowledge Engineering of Ministry of Education, Jilin University, Changchun, China; 3 College of Software, Jilin University, Changchun, China; Wuhan University of Science and Technology, CHINA

## Abstract

Three-dimensional (3D) image reconstruction is an important field of computer vision for restoring the 3D geometry of a given scene. Due to the demand for large amounts of memory, prevalent methods of 3D reconstruction yield inaccurate results, because of which the highly accuracy reconstruction of a scene remains an outstanding challenge. This study proposes a cascaded depth residual inference network, called DRI-MVSNet, that uses a cross-view similarity-based feature map fusion module for residual inference. It involves three improvements. First, a combined module is used for processing channel-related and spatial information to capture the relevant contextual information and improve feature representation. It combines the channel attention mechanism and spatial pooling networks. Second, a cross-view similarity-based feature map fusion module is proposed that learns the similarity between pairs of pixel in each source and reference image at planes of different depths along the frustum of the reference camera. Third, a deep, multi-stage residual prediction module is designed to generate a high-precision depth map that uses a non-uniform depth sampling strategy to construct hypothetical depth planes. The results of extensive experiments show that DRI-MVSNet delivers competitive performance on the DTU and the Tanks & Temples datasets, and the accuracy and completeness of the point cloud reconstructed by it are significantly superior to those of state-of-the-art benchmarks.

## Introduction

The purpose of 3D reconstruction is to recover the 3D geometry of a scene from a set of calibrated 2D images. With continual developments in computer vision [1], 3D reconstruction has emerged as one of the most important technologies for such applications such as medical imaging [2], Augmented Reality [3], and autonomous driving [4]. 3D geometry provides comprehensive information on the given scene in the above-mentioned fields. Multi-view stereo (MVS) reconstruction [5] is one of the most common methods of 3D reconstruction. It uses the known parameters of the camera and a set of images captured from multiple views of a given scene to generate the 3D reconstruction. The point cloud generated by MVS contains adequate environmental information, and thus it has been widely studied for decades.

Methods of MVS reconstruction can be categorized into the following types [6]: voxel-based [7], mesh-based [8], patch-based [9] and depth map-based methods [10, 11]. Voxel-based methods divide the scene into multiple voxels and determine whether they are located on the surface of the scene. However, they are limited in terms of reconstructing large-scale scenes owing to the high computational complexity of the voxels and the large amount of memory consumed. Mesh-based methods triangulate the scene to obtain rough triangle meshes and then reconstruct it by continuously deforming them. Patch-based methods extract the features of the image, transform them into patches, and match these patches with the 3D shape of the scene. However, patch-based methods require a robust filtering algorithm to eliminate incorrectly matched patches. Depth map-based methods reduce 3D reconstruction to multi-view depth map estimation. This significantly reduces temporal complexity by fusing multi-view depth maps into a point cloud. These methods are thus more flexible than the other methods above. Traditional and deep learning-based methods are the two main categories of methods of MVS reconstruction.

Traditional methods estimate the depth of pixels by using the projection relation between different views of the same scene. Tola et al. [12] proposed a method to reconstruct large-scale 3D scenes by using robust descriptors to significantly reduce the time needed. Galliani et al. [13] proposed a massively parallel method for MVS reconstruction called Gipuma, which uses a diffusion-like scheme to generate 3D planes in the scene space. Schonberger et al. [14] proposed COLMAP that uses hand-crafted features to optimize the depth value of each pixel. COLMAP performs well on public benchmarks and thus has become the standard for traditional MVS methods. While these methods have shown impressive results, they are still affected by changes in lighting, low-texture areas, and an unstructured viewing geometry. The resulting loss of low-level information leads to unreliable reconstruction.

Learning-based methods have shown significant potential as a replacement for traditional methods due to the robustness of the convolutional neural network (CNN) [15, 16]. Luo et al. [17] proposed an end-to-end learning network called P-MVSNet in which a patch-wise aggregation module is used to learn the correspondence-related information of the image features. However, the surface of the reconstructed scene represented by the mesh and the patch is not smooth, and details of the scene are thus lost. To recover more details of the scene, Xu et al. [18] proposed a lightweight method to measure the average group-wise correlation-based similarity. This can reduce the memory consumption as well as the computational burden and cost of filtering. Xiang et al. [19] proposed a network called PruMVS that utilizes a pruning strategy to reconstruct high-quality non-Lambertian surface and low-texture regions.

Although deep learning-based MVS methods [20, 21] have achieved excellent results in 3D reconstruction, they still have room for improvement in three areas. First, during feature extraction, it is important to capture the long-distance dependence of features in the channel-related and spatial dimensions; otherwise, a large amount of characteristic information is lost. Second, when calculating the cost of pixel matching in multi-view reconstruction, the weight representation of different pixels should be learned so that the model can adaptively calculate the cost of pixel matching to intuitively reflect the differences between pixels in different views. Third, given that the pixel parallax is inversely proportional to the depth, the interval between the depth planes in the cost volume should be gradually increased to enable a pixel to accurately find its matching pixel along the direction of the epipolar line.

This study proposes a cascaded deep network, DRI-MVSNet, with a cross-view similarity-based feature map fusion module for residual inference. A channel and spatial combined processing (CSCP) module is proposed to capture channel-related and spatial information of the feature map. Multi-view images are input into the feature extraction block to extract the feature maps. The feature maps are then entered into the CSCP module, which obtains channel-related information by using the channel attention mechanism. A spatial pooling network with different core shapes is used to collect spatial information. The CSCP module can thus capture large amounts of channel-related and spatial information to improve feature representation. The cross-view similarity-based feature map fusion (CVSF) module is proposed to intuitively reflect the difference in pixels between each source image and the reference image. In CVSF, a 2D similarity-weighted map is designed to learn the similarity of pixel pairs between each source feature map and the reference feature map, where this provides important information for depth map estimation from different views. At different depths, the reference feature map and each source feature map are concatenated on the channel to obtain a new feature map. Then, the concatenated feature map is convolved through the 3D convolution block and the sigmoid layer to obtain multiple 2D similarity-weighted maps from different views. Finally, to distribute the projected pixels of the source image uniformly along the direction of the epipolar line and improve the accuracy of the estimated residual depth, a multi-stage depth residual prediction (MDRP) module is proposed. It uses a non-uniform depth sampling strategy to construct hypothetical depth planes. The DRI-MVSNet is a three-stage cascade network that uses a coarse-to-fine strategy to refine the depth map. In the first stage, a rough depth map is obtained by using a uniform sampling strategy, and is used as the initial depth. Following this, the MDRP module is used to estimate the residual depth map in the next two stages. The depth map is refined iteratively through the residual depth map. Specifically in the MDRP module, a non-uniform depth sampling strategy is first used to construct hypothetical depth planes. The soft-argmin function is then used to obtain the probability value of each depth plane. The difference between the hypothetical depth planes and the ground truth depth value is then obtained, and is multiplied by the probability value to obtain the residual depth map. The refined depth map is obtained by adding the residual depth map and the initial depth map following up-sampling. Through these steps, DRI-MVSNet enhances the accuracy and completeness of 3D reconstruction.

The DRI-MVSNet was trained and tested on the DTU [22] and Tanks & Temple datasets [23]. The experimental results show that it achieves excellent performance competitive with state-of-the-art benchmarks.

The main contributions of this paper are as follows:

1. The CSCP module combines the channel attention mechanism and spatial pooling network to capture channel and spatial information of the scene.
2. The cross-view similarity-based feature map fusion module fuses multiple feature maps by introducing 2D similarity-weighted maps to reflect the similarity between each source image and the reference image for pixels at different depths.
3. The multi-stage depth residual prediction module can accurately predict the residual depth. This ensure that the hypothetical depth planes are constructed by using an effective and non-uniform depth sampling strategy.

The remainder of this paper is organized as follows: The next section introduces related work in the area. In the "Method" section, we describe our proposed DRI-MVSNet. Following this, the experimental results as well as a comparison between the proposed method and state-of-the-art methods is provided and discussed in the "Experiment" section. The final section summarizes the conclusions of this study.

## Related work

### SoTA methods

Table 1 summarizes state-of-the-art methods that have been recently applied to 3D reconstruction.

**Table 1:** Summary of recent methods for 3D reconstruction.

Method	Reference	Year	Single-view Reconstruction	Multiview Reconstruction
Voxel-based Methods	[26]	2021	Yes	No
	[25]	2021	No	Yes
	[24]	2021	No	Yes
Patch-based Methods	[17]	2019	No	Yes
	[27]	2021	No	Yes
	[28]	2021	No	Yes
Point cloud-based Methods	[29]	2020	Yes	No
	[30]	2021	Yes	No
	[31]	2021	Yes	No
Depth map-based Methods	[32]	2020	No	Yes
	[33]	2020	No	Yes
	[34]	2021	No	Yes

#### Voxel-based methods

Ji et al. [24] presented a volumetric method named SurfaceNet+ to handle sparse-MVS by introducing an occlusion-aware view selection scheme. Xie et al. [25] proposed a framework for reconstructing the three-dimensional shape of an object from a pair of stereo images. The framework restores the three-dimensional shape of the object by considering the bidirectional disparities and feature correspondences between the two views. Tahir et al. [26] presented a voxel- based three-dimensional object reconstruction method named V3DOR that utilizes automatic encoder (AE) and variational automatic encoder (VAE) to learn the appropriate compressed potential representation from a single 2D image.

Voxel-based methods consume a lot of GPU memory, which limits the reconstruction of this kind of method in large-scale scenes but can only be applied in small-scale scenes.

#### Patch-based methods

Luo et al. [17] proposed an end-to-end MVS network to build the matching cost volume. Through a patch-wise matching strategy, pixel-wise information can be learned from extracted features. Lv et al. [27] presented an MVS method of dense 3D road map is proposed, which integrates semantic information into the MVS pipeline based on patch matching. Stathopoulou et al. [28] presented an approach, which applies a priori knowledge to MVS based on patch matching to increase confidence and support depth and normal map estimation.

Patch-based methods often have the problem of insufficient reconstruction accuracy, which requires a very robust filtering strategy to ensure the reconstruction accuracy.

#### Point cloud-based methods

Li et al. [29] presented a network named 3D-ReConstnet that utilizes the residual network to extract the features of a 2D image to deal with the uncertainty of the self-occluded part of an object. Jin et al. [30] used an encoder-decoder framework to encode the RGB information in latent space and to predict the 3D shape of an object from multiple views. Chen et al. [31] presented a network named 3D-ARNet, which uses an encoder with an attention mechanism to extract image features and output point cloud.

Point cloud-based methods cannot achieve parallel computing, it greatly improves the time required for reconstruction, which leads to the fact that this method cannot achieve dense reconstruction in large-scale scenes.

#### Depth map-based methods

Luo et al. [32] presented a network named AttMVS that combines the raw pixel-wise matching confidence with the contextual information to improve the matching robustness. Xu et al. [33] proposed a network named PVSNet that learns the visibility information of different images. PVSNet uses the visibility information to construct an adaptive weighted cost volume. Weilharter et al. [34] presented HighRes-MVSNet, a network with a pyramid encoder-decoder structure searching for depth correspondences incrementally over a coarse-to-fine hierarchy.

The depth map-based method decouples 3D reconstruction into depth map estimation. By fusing multiple depth maps from different views into point clouds, high-precision 3D reconstruction can be realized and GPU memory consumption can be significantly reduced.

### 3D reconstruction

Three-dimensional reconstruction is the process of rebuilding the 3D geometry of a scene from a single view or multiple views. According to the number and characteristics of the vision sensors used, mainstream vision reconstruction systems include monocular [35], binocular stereo [36], and MVS [5] reconstructions. Monocular reconstruction uses one vision sensor to estimate the depth of each pixel in an RGB image, and requires only one image to restore the geometry of the scene. However, its application is limited owing to a lack of environmental information. A large amount of prior information is required to obtain good results of 3D reconstruction. The binocular stereo system is composed of two cameras based on the parallax principle. The 3D reconstruction of the scene is carried out by calculating the positional deviation between pixels in the left and right images. In binocular stereo systems, the corresponding pixels are difficult to restore in occluded areas and areas with low texture, and this limits their application. MVS based on 3D CNN uses three or more cameras to capture a series of images from different views to restore the 3D scene. MVS contains a wealth of scene-related information and a larger field of vision, which solves the problem of ambiguity of binocular stereo matching and improves the accuracy of reconstruction. However, 3D CNNs require a large amount of GPU memory, and prevalent MVS methods struggle to achieve adequate reconstruction while consuming a small amount of memory. In this study, we use a coarse-to-fine strategy to enhance spatial resolution and reduce the range of depth, and reduce memory consumption by constructing a cascaded cost volume.

### Multi-view stereo reconstruction

Multi-view stereo reconstruction is a 3D reconstruction algorithm that involves the use of a collection of images and the corresponding parameters of the camera to output representation of a scene based on one of the following: voxel s, patches, point clouds, and depth maps. In general, voxel-based methods discretize the 3D space into a 3D grid and determine whether a given voxel is on the surface. However, they are usually limited by the resolution of the scene. Patch-based methods first initialize the image into triangular meshes and then continuously deform them to output the mesh representation of the scene. However, patch-based methods are not convenient for dealing with objects with irregular topologies. Point cloud-based methods first sample a series of key points and then rely on the propagation strategy to densify the point cloud. As the propagation of point clouds proceeds sequentially, point cloud-based methods usually take a long time to process. Depth map-based methods construct the cost volume by using plane-sweeping approaches and further generate depth maps from different views. By contrast, our proposed procedure to predict the depth residual estimates the depth offset of each pixel by learning the probability distribution of the non-uniform depth plane in the direction of depth to iteratively optimize the depth map.

### Learning-based MVS

Recent learning-based approaches have shown significant potential for use in MVS reconstruction. One such approach is based on the volumetric representation of the surfaces of scenes. Ji et al. [37] proposed an end-to-end learning framework named SurfaceNet that voxelizes the solution space and predicts whether the voxels are on the surface of the scene by constructing colored voxel cubes. Kar et al. [38] proposed an end-to-end learnable system named LSM in which differentiable projection and a lack of projection are used to infer the underlying 3D shape. Another strategy uses the plane-sweep stereo to build the matching cost volume and then the depth maps to represent the scene. MVSNet [39] is a pioneering method that generates point clouds from multi-view images, constructs the cost volume using homographic warping, and then acquires depth maps via a soft argmin operation. Xue et al. [40] proposed a deep-learning multi-view stereo architecture with conditional random fields called MVSCRF. It first extracts features incorporating both local and global information by using a U-shaped network, and then constrains smoothness via the conditional random fields. While these methods have delivered impressive results, their efficiency is still far from satisfactory. We propose a cascaded network with a cross-view similarity-based feature map fusion module to reflect the differences in pixels between each source image and the reference image to infer the residual depth, where better features that are better matched element wise can be enhanced while mismatch errors can be reduced.

### Attention mechanism

The attention mechanism is a data processing method in machine learning that can obtain detailed useful information and suppress useless information. Hu et al. [41] introduced an attention mechanism named SENet that adaptively computes channel-wise feature responses by explicitly modeling the interdependencies between channels. They used pooled, average global features to recalibrate channel-wise attention. However, they ignored spatial information, which plays an important role in image recognition. Woo et al. [42] proposed an attention module named CBAM to infer attention maps along the dimensions of the channel and space. In contrast to SENet, CBAM utilizes the positional relationship between features to obtain a spatial attention map. Fu et al. [43] proposed a dual attention network (DANet) based on the self-attention mechanism to adaptively combine local features with their global dependencies. DANet selectively aggregates dependencies between each feature and other features in the spatial and channel-related dimensions. Hou et al. [44] proposed a pooling strategy called strip pooling that reconsiders the formulation of spatial pooling by constructing a kernel of size 1 × *N* or *N* × 1.

Feature extraction is a key issue in MVS reconstruction. Current MVS-based methods cannot capture long-range dependencies in the spatial and channel-related dimensions. To address these issues, we introduce a channel and spatial attention mechanism to the feature extraction procedure to improve the overall of completeness of the results of reconstruction.

### Cost volume

The cost volume is a measure of the quality of matching between features. It uses a standard metric to reflect the cost of matching multiple input images on each hypothetical depth plane. The lower the cost value is, the better is the quality of the matching. Therefore, calculating the cost volume is essential for estimating the depth of the scene. Most MVS methods use plane-sweeping-based approaches to calculate the cost of stereo matching, but experiments have shown that cost calculations based on depth features are more robust against fuzzy regions. In many traditional stereo matching networks, images with a fixed resolution are used to construct cost volumes while high-resolution images lead to the consumption of a large amount of memory. Yang et al. [45] proposed a hierarchical cascading network in which the cost volume is constructed in a coarse-to-fine manner. In contrast to a previously proposed cost volume based on an interval with a fixed depth, we construct a non-uniform depth plane using a cascaded cost volume to obtain a more accurate cost of pixel matching.

## Method

**Fig 1:**
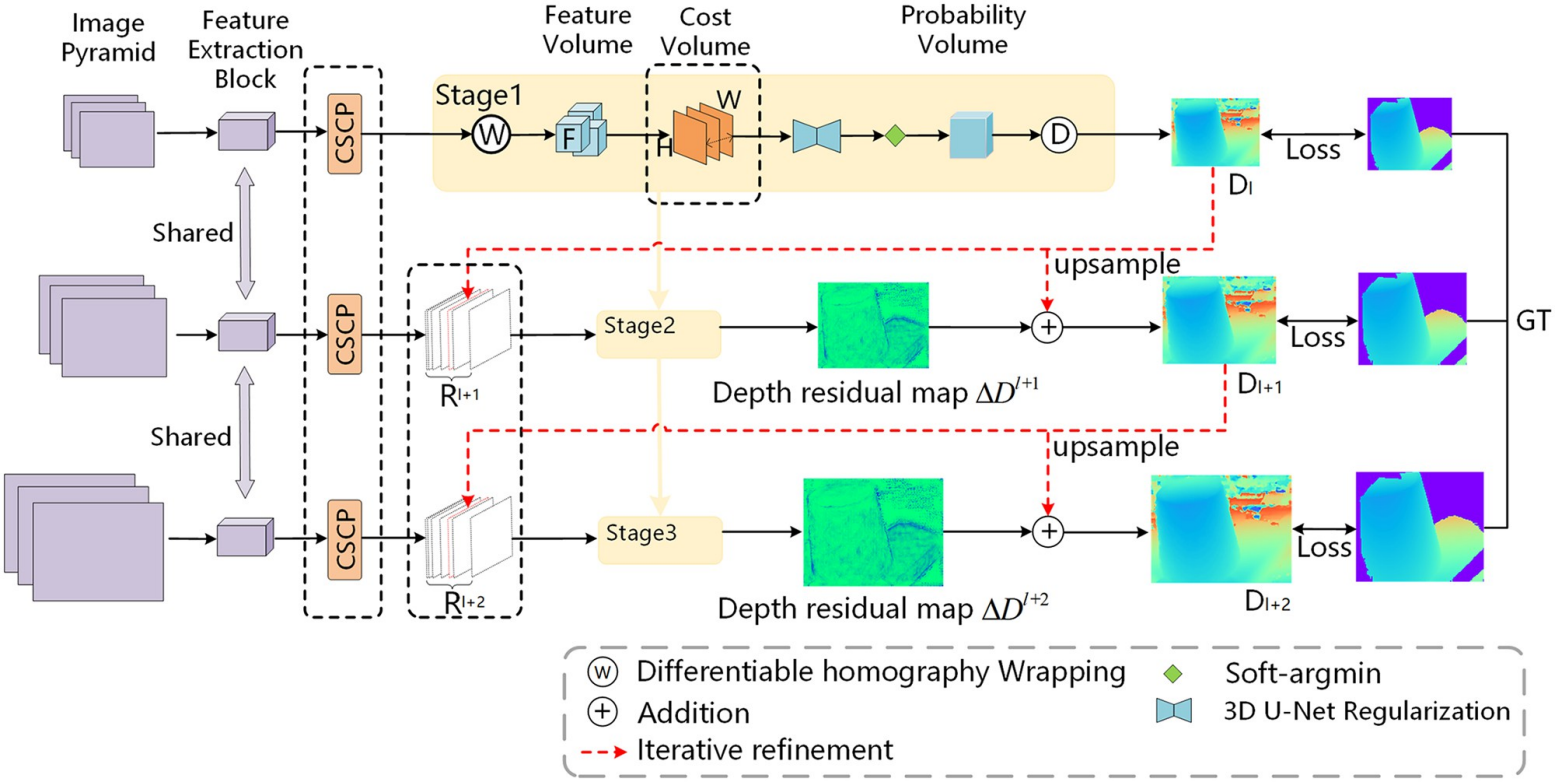
The network structure of DRI-MVSNet.

Given multi-view images and the corresponding parameters of the camera, the goal of DRI-MVSNet is to infer the depth map of the reference image to carry out 3D reconstruction. DRI-MVSNet is a network that consists of three parts: a channel and spatial combined processing module, a cross-view similarity-based feature map fusion module, and a multi-stage depth residual prediction module. The overall structure of the proposed DRI-MVSNet is shown in Fig 1. It is a three-stage cascade network that generates high-precision depth maps in a coarse-to-fine manner. In each stage, DRI-MVSNet first inputs the multi-view feature maps to the CSCP module to process each feature in the channel-related and spatial dimensions. The cross-view similarity-based feature map fusion module is then used to warp multiple feature maps into a cross-view cost volume. The depth map is generated through the soft-argmin layer, and the map obtained in the first stage is regarded as the initial depth map. While the hypothetical depth planes in the first stage are constructed in a uniform manner, those in the other stages are constructed by using a non-uniform depth sampling strategy. The residual depth maps of the second and third stages are obtained by using the multi-stage depth residual prediction module. The residual depth map is used to iteratively refine the initial depth map. In the following, each component is introduced in detail.

### Feature extraction using image pyramid

The image pyramid is used to extract features from images with different resolutions to reduce the amount of required memory. Given a reference image *I*_1_ ∈ *R*^*H*×*W*×3^, a set of source images is defined as in Eq (1):

$$S=\left\{I_{i}\in R^{H\times W\times 3}|i=2,\ldots ,N\right\},$$ (1)

where *H* and *W* are the height and width of the image, respectively, and *N* is the number of input images. Multi-view images are down-sampled to build a three-level image pyramid *Q*, which is defined as in Eq (2):

$$Q=\left\{I_{i}^{l}\in R^{H/2^{3-l}\times W/2^{3-l}\times 3}|i=1,\cdots ,N,l=1,2,3\right\},$$ (2)

where *l* is the number of levels of the image pyramid. A feature extraction module with shared parameters is used to extract the features of each level of the image. It is composed of nine convolutional layers, each of which is followed by a leaky rectified linear unit.

For each input image $I_{i}^{l}$, the feature extraction module extracts feature maps *F*, which are defined as in Eq (3):

$$F=\left\{f_{i}^{l}\in R^{H/2^{3-l}\times W/2^{3-l}\times C^{l}}|i=1,\cdots ,N,l=1,2,3\right\},$$ (3)

where *C*^*l*^ is the number of channels of the feature map of the l-th layer. Each image *I*_*i*_ has three feature maps of sizes $\frac{H}{4}\times \frac{W}{4}$, $\frac{H}{2}\times \frac{W}{2}$, and *H* × *W*, and they have 32, 16, and 8 channels, respectively. The extracted features are then input to the CSCP module for feature enhancement processing. Compared with many available methods, the introduction of the image pyramid-based feature extraction module significantly improves the accuracy of scene reconstruction.

### Channel and spatial combined processing module (CSCP)

**Fig 2:**
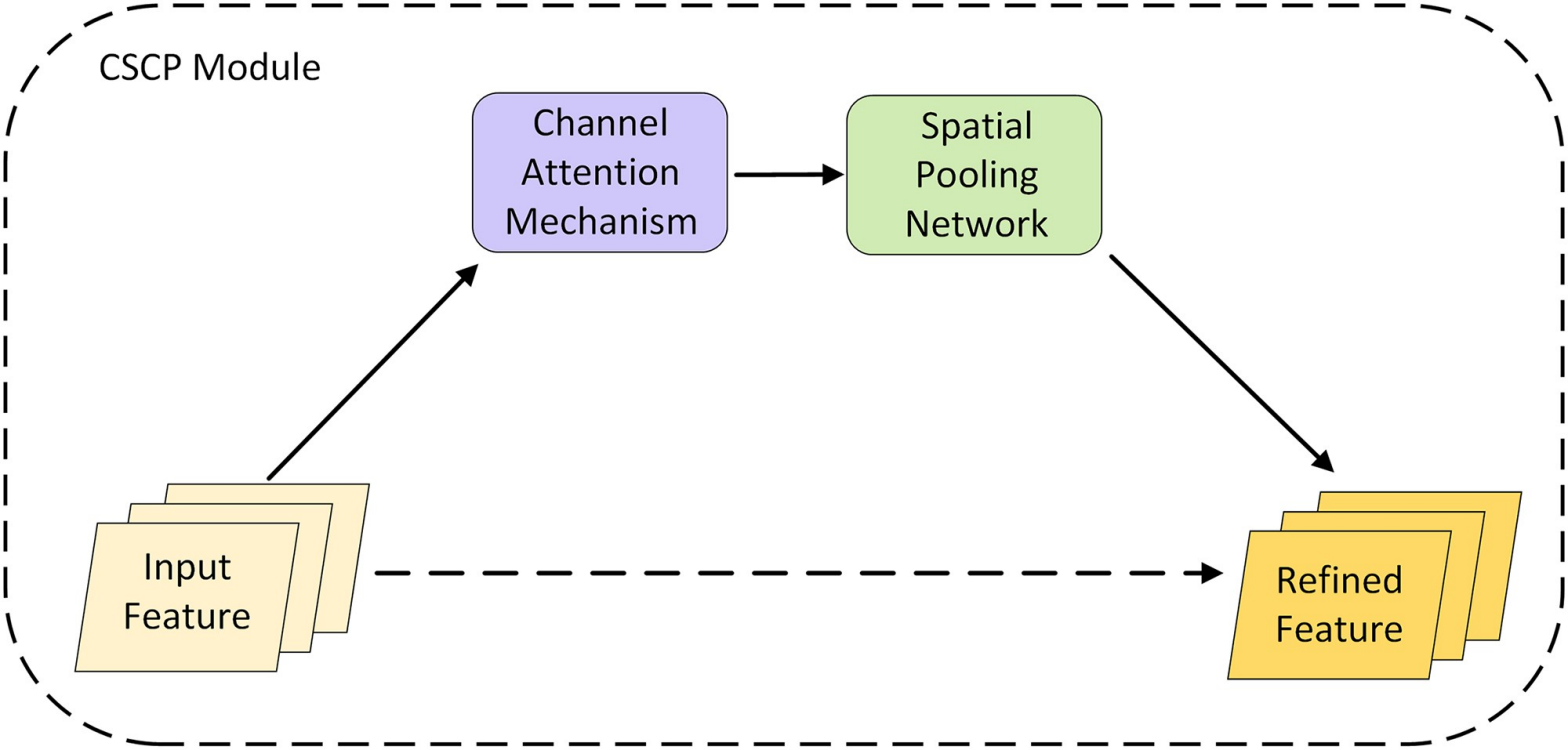
The channel and spatial combined processing (CSCP) module.

In the field of 3D reconstruction, excellent feature recognition is essential for understanding a given scene. Inspired by the work in Ref. 40, semantic interdependence is modeled in the channel-related and spatial dimensions in the feature extraction stage to obtain a rich feature representation. The CSCP is composed of a channel attention mechanism and a spatial pooling network, as shown in Fig 2. The feature maps extracted by the feature extraction network are entered into the CSCP module, where the channel attention mechanism helps the model obtain information on useful features, while the spatial pooling network encodes the long-range context along the horizontal and vertical spatial dimensions. The CSCP captures the long-distance dependencies of features as well as aggregates global and local context information. Compared with other methods, such as directly using the original image to extract deep features, CSCP is more robust against low-texture regions.

#### Channel attention network

**Fig 3:**
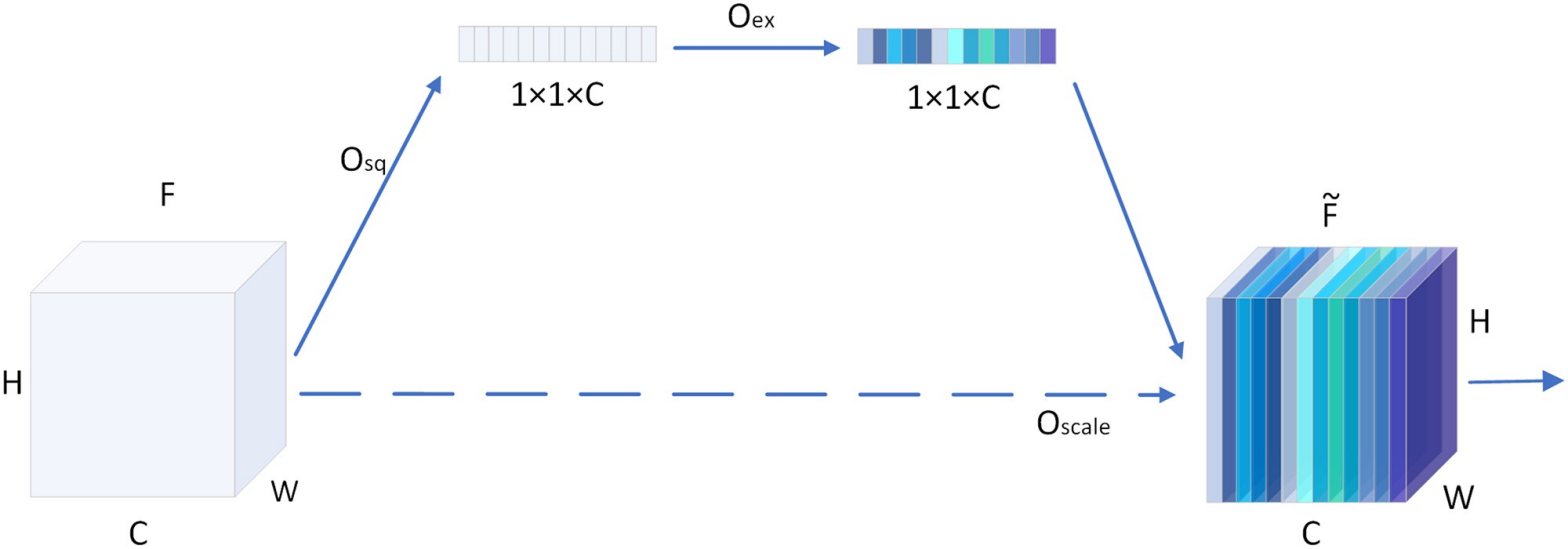
Illustration of SENet.

The channel attention network is a common unit in deep learning networks that is used to explicitly model the interdependence between channels and adaptively learn the contribution of each channel. SENet is a channel attention network that is widely used in the field of feature recognition. SENet is embedded into the CSCP to improve the quality of feature representation as shown in Fig 3. Its implementation consists of two parts: a squeeze operation and an excitation operation. To simplify the formula, *l* is omitted. We use the squeeze operation *O*_*sq*_ on the feature map to compress the global spatial information into the channel descriptor *z* through global average-pooling such that the c-th element of *z*_*c*_ is defined as in Eq (4):

$$z_{c}=O_{sq}(f_{i,c})=\frac{1}{H\times W}\sum_{a=1}^{H}\sum_{b=1}^{W}f_{i,c}(a,b),$$ (4)

where *f*_*i*,*c*_ is the feature of *f*_*i*_ on the c-th channel. Then, the excitation operation *O*_*ex*_ is used to fully capture the dependencies of the channels; it consists of two fully connected layers as well as ReLU and sigmoid layers, and is used to generate the channel weight *s*_*c*_, which is defined as in Eq (5):

$$s_{c}=O_{ex}(z_{c},W_{1},W_{2})=\sigma (W_{2}\delta (W_{1}z_{c})),$$ (5)

Where *δ* is the ReLU function, *σ* is the sigmoid function, and *W*_1_ and *W*_2_ are the fully connected layers. We rescale *f*_*i*,*c*_ by activating *s*_*c*_ to get the final output ${\tilde{f}}_{i,c}$, which is defined as in Eq (6):

$${\tilde{f}}_{i,c}=O_{scale}(f_{i,c},s_{c}),$$ (6)

Where *O*_*scale*_(·,·) is channel-wise multiplication of the feature map *f*_*i*,*c*_ and the weighted score *s*_*c*_, and $\tilde{F}=\left[{\tilde{f}}_{1,c},{\tilde{f}}_{2,c},\ldots ,{\tilde{f}}_{i,c}\right]$.

#### Spatial pooling network

**Fig 4:**
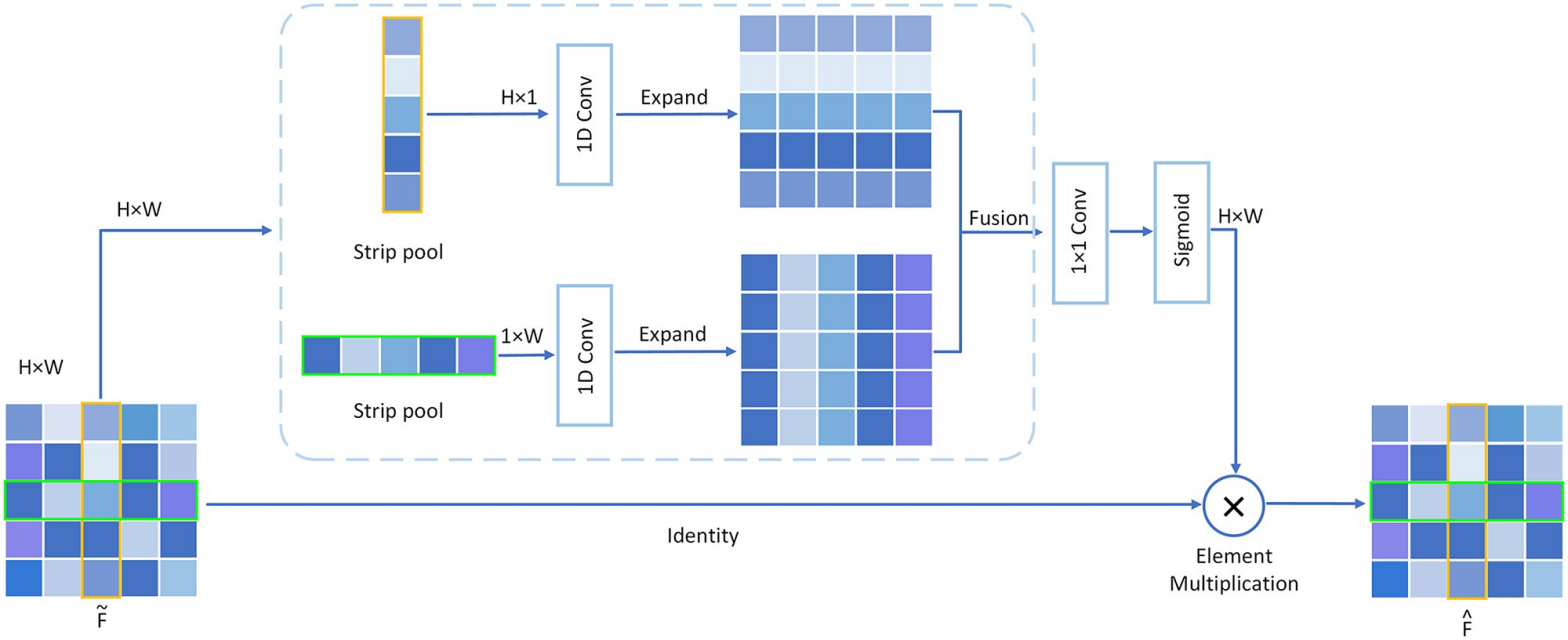
Illustration of strip pooling network.

The method based on spatial pooling to capture spatial contextual information has made extraordinary progress in scene parsing. The strip pooling module (SPM) [44] is a lightweight spatial pooling network that builds long-range dependencies from different spatial dimensions. SPM is directly embedded into the CSCP behind the SENet, where this makes up for the insufficient ability of SENet to capture spatial contextual information. SPM accomplishes spatial pooling by using a band-shaped pooling window along the horizontal and vertical dimensions to build the long-range dependencies of discrete areas of distribution. The SPM is shown in Fig 4. Given ${\tilde{f}}_{i}$ as the input to the SPM, we first input ${\tilde{f}}_{i}$ into two pathways, each of which contains a horizontal or vertical strip pooling layer that averages all elements in a row or a column. A 1D convolutional layer with a kernel size of three follows, and *y*^*h*^ ∈ *R*^*C×H*^ and *y*^*v*^ ∈ *R*^*C×W*^ are then obtained. We then combine *y*^*h*^ and *y*^*v*^ to get the result y, which is defined as in Eq (7).

$$y=y^{h}+y^{v}.$$ (7)

Finally, we input *y* into the 1 × 1 convolutional layer and the sigmoid layer. The final output $\hat{F}$ is defined as in Eq (8):

$$\hat{F}=\left\{{\hat{f}}_{i}=Scale({\tilde{f}}_{i},\sigma (\tau (y)))|i=1,\ldots ,N\right\},$$ (8)

Where *Scale*(·,·) is the element-wise multiplication of ${\tilde{f}}_{i}$ and *σ*(*τ*(*y*)), *σ* is the sigmoid function, and *τ* is a 1 × 1 convolution. Local information is also captured owing to the long and narrow kernel. The SPM is a lightweight network module that yields good performance. It can be considered a spatial attention mechanism owing to the element-wise multiplication operation.

### Feature volume construction

Given the feature maps and range of depth, we construct the cost volume. The range of depth search of each pixel is first determined to construct fronto-parallel planes. Then, the feature maps of all views are warped along the direction of the frustum of the camera to form the feature volumes. Finally, the CVSF module is used to fuse the feature volumes to generate the cost volume. Note that the CVSF can adaptively learn the weights of different feature volumes to calculate the cost of the pixels.

The feature volume is constructed for the reference image and each source image. In order to perform a homography warp on the feature map, *M* parallel hypothetical planes are constructed. While the hypothetical depth planes in the first stage are constructed in a uniform manner, those of the other stages are constructed by non-uniform depth sampling. Given the range of depth [*d*_min_, *d*_max_] of the reference images, *d*_min_ and *d*_max_ are the minimum and the maximum depths, respectively, that are evenly divided into *M* parallel hypothetical planes that are defined as in Eq (9):

$$d_{m}=d_{\text{min}}+\frac{m(d_{\text{max}}-d_{\text{min}})}{M},m=1,\cdots ,M,$$ (9)

where *m* is the index of the hypothetical depth planes.

In order to warp the feature map into the feature volume, the differentiable homography matrix is defined as in Eq (10):

$$H_{i}(d_{m})=K_{i}^{l}R_{i}(E-\frac{(t_{1}-t_{i}){\vec{n}}_{1}^{T}}{d_{m}})R_{1}^{-1}{\left(K_{1}^{l}\right)}^{-1},$$ (10)

Where *H*_*i*_(*d*_*m*_) is the homography matrix between the i-th source feature map and the reference feature map at depth, *E* is the identity matrix, $K_{i}^{l}$, *R*_*i*_, and *t*_*i*_ are the intrinsic matrix of the camera, the rotation matrix, and the translation vector, respectively, and ${\vec{n}}_{1}^{T}$ is the unit length normal vector of the plane.

All feature maps are then warped into parallel hypothetical planes of the reference image by using the homography matrix to form a feature volume set *V*, which is defined as in Eq (11):

$$V=\left\{V_{i}=CONCAT(\frac{H_{i}(d_{m}){\hat{f}}_{i}}{d_{m}})|i=1,\ldots ,N,m=1,\ldots ,M\right\},$$ (11)

Where *CONCAT*() is the concatenation operation of the warped feature map along the direction of depth.

### Cross-view similarity-based feature map fusion module (CVSF)

**Fig 5:**
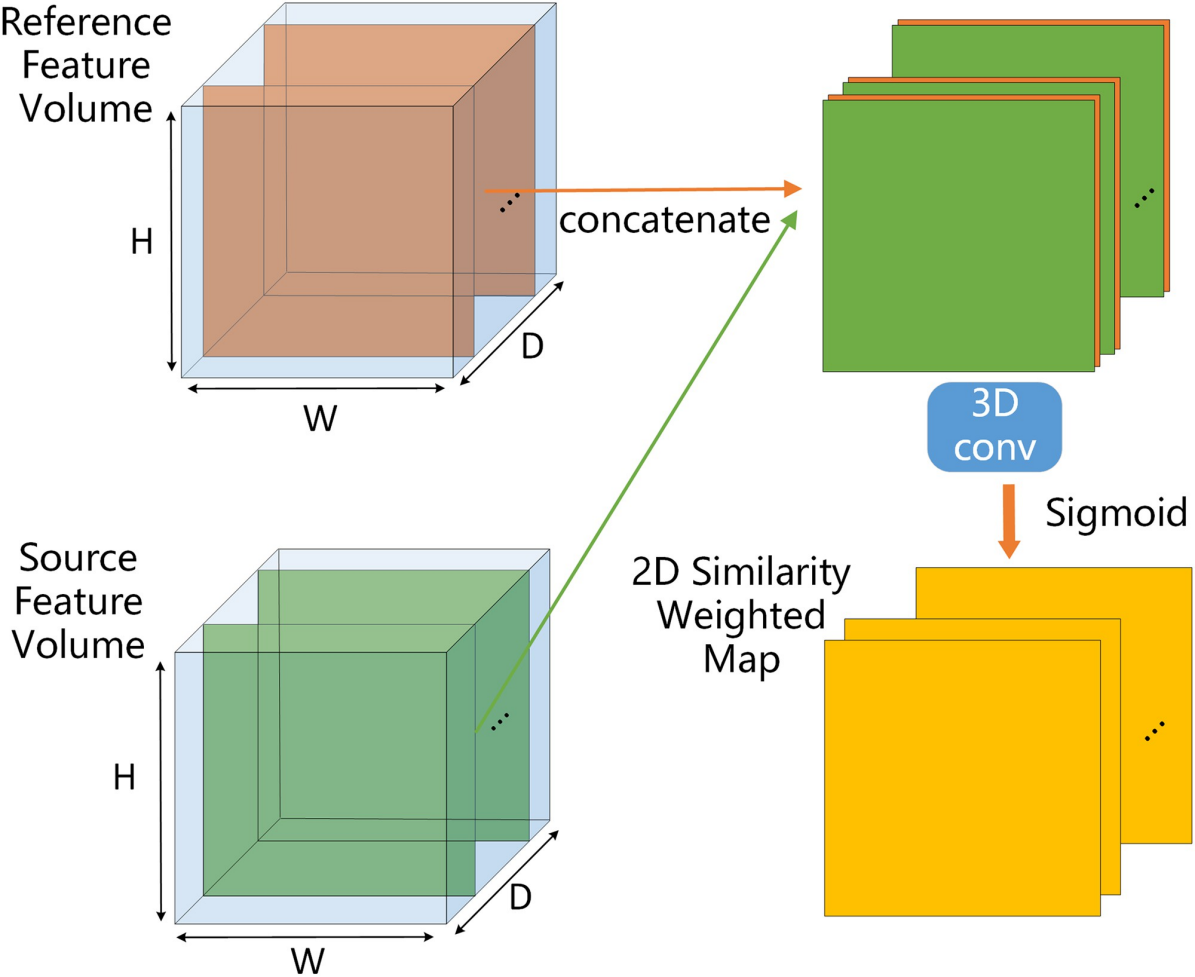
Illustration of cross-view similarity-based feature map fusion module.

This section describes how the set of feature volumes *V* is aggregated into a cross-view cost volume. Note that the key to the CVSF is to allow the network to adaptively learn the similarity between pixel pairs at different depths for each source image, as shown in Fig 5. A similarity-weighed map is used to represent the pixel-wise degree of matching between each source image and the reference image. The formula yields the smallest squared difference in features for each pixel under the correctly estimated depth. The depth dependence of pixels in local regions is encoded by CNNs. At different depths, the CVSF concatenates the reference feature map $V_{1,d_{m}}$ and the source feature map $V_{i,d_{m}}$ at depth *d*_*m*_ on the channel. The concatenated feature maps are then convolved through the 3D convolution block and the sigmoid layer to obtain the 2D similarity-weighted map $w_{i,d_{m}}$.

Given the feature volume *V*_*i*_ and the corresponding 2D similarity-weighted map $w_{i,d_{m}}$, the cost *C*(*d*_*m*_, *q*) of pixel *q* at depth *d*_*m*_ is defined as in Eq (12):

$$C(d_{m},q)=\frac{\sum_{i=2}^{N}{\left(V_{i,d_{m}}-V_{1,d_{m}}\right)}^{2}\cdot w_{i,d_{m}}}{N},$$ (12)

where $V_{i,d_{m}}$ is the feature map of *V*_*i*_ at depth *d*_*m*_.

The CVSF intuitively reflects the difference in feature-related information between the source images and the reference images, and transfers the absolute feature representation to the cost volume. The introduction of the similarity-weighted map helps control the relative influence of each pixel and significantly improves the accuracy of estimation of the cost value.

### Estimating the coarse depth map

We use the estimated depth of the image with the coarsest resolution as the initial depth. The MDRP is then introduced. According to it, the residual prediction refines the depth map iteratively to achieve a more accurate depth inference. The MDRP module is mainly used in the latter two stages of the network.

Owing to a lack of texture in the image, the cost volume inevitably contains noise. To filter out noise in the cost volume, the 3D convolution is used to regularize it. The probability volume *P* is then obtained by executing the soft-argmin operation on the cost volume *C*. *P* is a probability volume composed of multiple probability planes, where the value of each pixel represents the probability that the pixel is located in a certain plane. The depth of pixel *q* in the coarse depth map is defined as in Eq (13):

$$D^{l}(q)=\sum_{m=1}^{M}d_{m}P_{q}^{l}(d_{m}),$$ (13)

where $P_{q}^{l}(d_{m})$ is the probability that pixel *q* is on the m-th virtual depth plane.

### Multi-stage depth residual prediction module (MDRP)

The network estimates depth maps in a rough-to-fine manner, and a coarse depth map obtained in a given stage is used as a prior for the next stage. We start iterating from the first stage, with the depth map *D*^*l*^ as the initial depth map. The MDRP module is used to estimate the depth residual Δ*D*^*l*+1^ in stage *l* + 1. We then upsample *D*^*l*^ to the next level $D_{↑}^{l}$ via bicubic interpolation, and then use $D^{l+1}=D_{↑}^{l}+\Delta D^{l\text{+1}}$ to refine the depth for high-density depth inference. In contrast to Ref. 24, the non-uniform depth sampling strategy is used in place of uniform sampling.

We use take the depth value *D*^*l*^(*q*), estimated in the l-th stage, as a benchmark, and determine the range of depth search *R*^*l*+1^ of pixel *q* for the next stage by using the standard deviation *σ*^*l*^(*q*), defined as in Eq (14).


$$\sigma^{l}(q)=\sqrt{\sum_{m=1}^{M}P_{m}^{l}(q)\cdot {\left(d_{m}^{l}-D^{l}(q)\right)}^{2}}.$$ (14)

Given the predicted depth *D*^*l*^(*q*) and its standard deviation *σ*^*l*^(*q*), a range of depth search *R*^*l*+1^ based on the standard deviation to measure uncertainty in the prediction in the next stage. This is defined as in Eq (15):

$$R^{l+1}=[D^{l}(q)-\sigma^{l}(q),D^{l}(q)+\sigma^{l}(q)].$$ (15)

**Fig 6:**
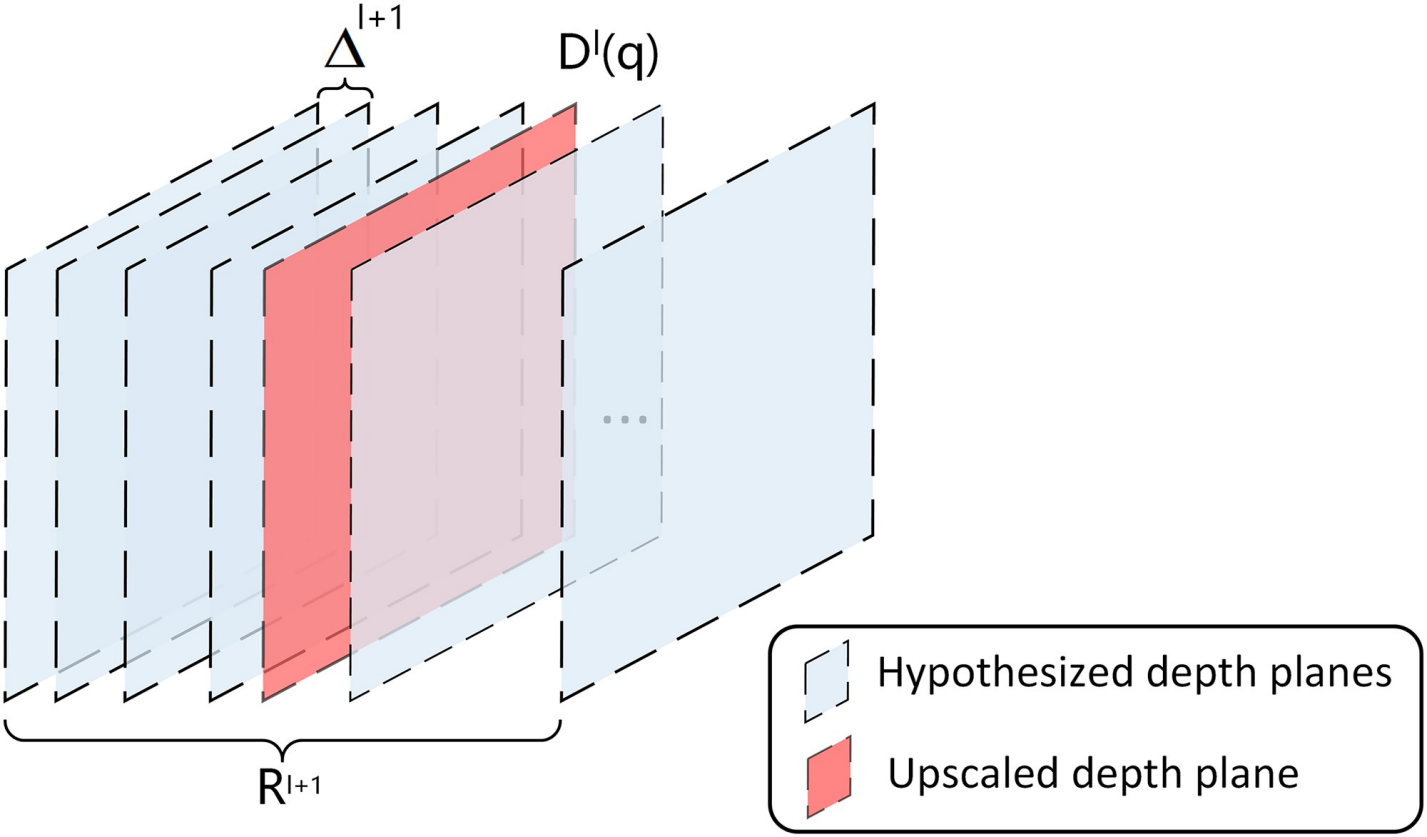
The non-uniform depth sampling strategy. *R*^*l*+1^ is the total range of search and Δ^*l*+1^ is the initial depth interval. The number of hypothetical depth planes M at different stages is 64, 32, and 8.

The non-uniform depth sampling strategy is used in the second and third stages to construct hypothetical depth planes, as shown in Fig 6. We construct an initial depth interval Δ^*l*+1^, which is defined as in Eq (16):

$$\Delta^{l\text{+1}}=\frac{R^{l\text{+1}}}{\sum_{i=1}^{M}r^{i-1}},$$ (16)

where *r* is the interval ratio, set to 1:2. Then, the non-uniform hypothetical depth planes are defined as in Eq (17):

$$d_{m}^{l+1}=d_{\text{min}}+\Delta^{l\text{+1}}\cdot \sum_{i=1}^{m}r^{i-1},m=1,\cdots ,M$$ (17)

The depth residual in the next stage is estimated to further refine the depth map. Precisely speaking, there is a relative dependency between each non-uniform hypothetical depth plane in a given stage and the depth map of the previous stage. The refined depth map is defined as in Eq (18):

$$D^{l+1}(q)=D_{↑}^{l}(q)+\sum_{m=1}^{M}\left(d_{m}^{l+1}-D^{l}(q))\cdot P_{m}^{l\text{+1}}(q\right)$$ (18)

### Loss function

Similar to previous methods [39], we use the differences between the ground truth and the estimated depth to train the proposed DRI-MVSNet. The training loss is defined as in Eq (19):

$$Loss=\sum_{l=1}^{L}\sum_{q\in \Omega }||D^{l}(q)-D_{GT}^{l}(q)|{|}_{1},$$ (19)

where Ω is the set of valid pixels with ground truth measurements and $D_{GT}^{l}(q)$ is the ground truth depth of the *l*-th stage.

## Experiment

In this section, we first introduce the DTU [22] and Tanks & Temples [23] datasets used to validate DRI-MVSNet. We then explain the experimental setup and evaluation metrics used to compare our method with prevalent methods in the area. To verify the effectiveness of the components of DRI-MVSNet, we conducted ablation experiments. Finally, we discuss reasons for why the proposed approach achieves better results than available methods.

### Datasets

#### DTU dataset

The DTU dataset is a large dataset of multi-view stereo images of 124 scenes. It contains 49 or 64 images captured under seven lighting conditions, each with an image of size 1600 × 1200 pixels. We followed the method provided by MVSNet to complete the experimental configuration. With the same size of the training, validation, and evaluation sets, a total of 27,097 images were used for DRI-MVSNet training.

#### Tanks & Temples dataset

Tanks & Temples benchmark contains a large number of images of outdoor scenes. Owing to such changeable natural conditions as lighting and reflection of the scene, the dataset is more complicated than the DTU. It contains eight scenarios: Family, Francis, Horse, Lighthouse, M60, Panther, Playground, and Train. We used these datasets to verify the generalization ability of DRI-MVSNet.

### Implementation details

#### Training

We trained our DRI-MVSnet on the DTU dataset. The entire dataset was split into a training set, a validation set, and an evaluation set. For a fair comparison, we trained our model on the DTU dataset according to the training configuration of MVSNet. The image resolution of the DTU dataset was 640 × 512. To facilitate training, we cropped the image to get an image of size 320 × 256. We built a three-level image pyramid for which the number of views *N* = 3. The depth hypothesis of the first stage involved sampling uniformly between 425 mm and 933.8 mm, and the number of depth planes acquired in the first to the third stages were 64, 32, and 8, respectively, and the sizes of the corresponding depth maps were $\frac{H}{4}\times \frac{W}{4}$, $\frac{H}{2}\times \frac{W}{2}$, and *H* × *W*, respectively. The model trained on the DTU training set was used to reconstruct the point cloud on the intermediate set of Tanks & Temples to evaluate the generalization ability of DRI-MVSNet, which was trained on PyTorch. DRI-MVSNet was optimized over 16 epochs by the Adam optimizer on a single NVIDIA GTX 2080Ti GPU. The initial learning rate was set to 0.001, and was then divided by two in the 10th, 12th, and 14th epochs. The batch size was set to four.

#### Evaluation metrics

We evaluated the reconstruction performance of the proposed method on the DTU and the Tanks & Temples datasets in terms of accuracy, completeness, overall score, and the F1-score of the point cloud reconstruction. Accuracy refers to the distance between the estimated point clouds and the ground truth, and completeness represents the distance between the ground truth and the estimated point clouds. For the DTU, the overall score refers to the average of the accuracy and completeness. For the Tanks & Temples dataset, the F1-score is the overall score of the measured percentage metric, and a higher F1-score reflects a better result.

### Results on DTU dataset

We first evaluated the accuracy, completeness, and overall score of reconstruction on the DTU dataset. Our results were compared with those of traditional geometry-based methods and other learning-based benchmark methods. As summarized in Table 2, DRI-MVSNet was superior to the other methods in terms of completeness and overall score. Among the traditional geometry-based methods, Gipuma delivered the best results in terms of accuracy. The accuracy of MVSCRF was superior to that of all learning-based methods.

**Table 2:** Quantitative results on the DTU dataset (lower is better).

Method	Acc.	Comp.	Overall
Camp [46]	0.835	0.554	0.695
Furu [47]	0.613	0.941	0.777
Tola [12]	0.342	1.190	0.766
Gipuma [13]	**0.283**	0.873	0.578
Colmap [14]	0.400	0.664	0.532
MVSNet [39]	0.396	0.527	0.462
SurfaceNet [37]	0.450	1.040	0.745
MVSNet [17]	0.406	0.434	0.420
R-MVSNet [48]	0.383	0.452	0.417
PruMVSNet [19]	0.495	0.433	0.464
MVSCRF [40]	0.371	0.426	0.398
AttMVS (M = 256) [32]	0.412	0.397	0.403
PVSNet (Low-Res) [33]	0.408	0.393	0.4001
SurfaceNet+ [24]	0.385	0.448	0.416
DRI-MVSNet (Ours)	0.432	**0.327**	**0.379**

### Results on Tanks & Temples dataset

We evaluated the generalization ability of DRI-MVSNet on the Tanks & Temples dataset in terms of the F1-score. The results of eight scenes are reported in Table 3. For most scenes, our method showed good results, with the best average F1-score of all methods tested. On the DTU dataset, the MVSCRF method had the best accuracy among all learning-based methods, but its average F1-score on the Tanks & Temples Dataset was 6.98% lower than that of our method. It can be seen that DRI-MVSNet achieves competitive results in most scenarios except for M60, Panther and Lighthouse. MVSNet++ realizes depth estimation in long-distance scenes by constructing more virtual depth planes (the number of planes is 256). This strategy is helpful for scenes with large depth range such as M60 and Panther. Generally speaking, the number of depth planes is positively correlated with the experimental results. SurfaceNet+ utilizes an occlusion-aware view selection scheme that takes the geometric prior into account. This strategy is more suitable for buildings with a large number of views such as Lighthouse because it considers the visibility of pixels.

**Table 3:** Quantitative results on Tanks & Temples benchmark (higher is better).

Method	Mean	Family	Francis	Horse	Lighthouse	M60	Panther	Playground	Train
MVSNet [39]	43.48	55.99	28.55	25.07	50.79	53.96	50.86	47.9	34.69
R-MVSNet [48]	48.4	69.96	46.65	32.59	42.95	51.88	48.8	52	42.38
Point-MVSNet [49]	48.27	61.79	41.15	34.24	50.79	51.97	50.85	52.38	43.06
VA-Point-MVSNet [50]	48.7	61.95	43.73	34.45	50.01	52.67	49.71	52.29	44.75
MVSNet++ [51]	49.12	62.64	38.49	39.60	48.40	**54.95**	**51.69**	52.28	44.92
MVSCRF [40]	45.73	59.83	30.6	29.93	51.15	50.61	51.45	52.60	39.68
Fast-MVSNet [52]	47.39	65.18	39.59	34.98	47.81	49.16	46.20	53.27	42.91
HighRes-MVSNet [34]	49.81	66.62	44.17	30.84	55.13	53.20	50.32	55.45	42.73
SurfaceNet+ [24]	49.38	62.38	32.35	29.35	**62.86**	54.77	54.14	56.13	43.10
DRI-MVSNet (Ours)	**52.71**	**73.64**	**53.48**	**40.57**	53.90	48.48	46.44	**59.09**	**46.10**

### Ablation studies

In this section, we report extensive ablation studies to evaluate the advantages and limitations of key components of DRI-MVSNet, including the CSCP module, cross-view similarity-based feature map fusion module, and multi-stage depth residual prediction module. All results were obtained on the DTU dataset and the Tanks & Temples dataset. Accuracy and completeness were used to measure the quality of reconstruction on DTU dataset and the F1-score was used to measure that on the Tanks & Temples dataset. The results are shown in Tables 4 and 5.

**Table 4:** Ablation study of the CSCP, CVSF, and MDRP on the DTU dataset.

Experiment	Model	Acc.	Comp.	Overall
Ablation study of the CSCP	CVSF+MDRP+baseline (without CSCP)	0.436	**0.324**	0.380
Ablation study of the CVSF	CSCP+MDRP+baseline (without CVSF)	0.435	0.338	0.387
Ablation study of the MDRP	CSCP+CVSF+baseline (without MDRP)	0.549	0.415	0.482
DRI-MVSNet	CSCP+CVSF+MDRP+baseline	**0.432**	0.327	**0.379**

**Table 5:** Ablation study of the CSCP, the CVSF and the MDRP on the Tanks & Temples dataset.

Experiment	Mean	Family	Francis	Horse	Lighthouse	M60	Panther	Playground	Train
Ablation study of the CSCP	48.06	67.39	50.01	37.10	50.25	41.69	**47.60**	48.35	42.07
Ablation study of the CVSF	49.12	69.30	53.35	35.72	51.22	48.02	44.53	51.73	39.10
Ablation study of the MDRP	42.78	57.12	48.63	29.74	46.39	39.32	35.37	45.21	40.42
DRI-MVSNet	**52.71**	**73.64**	**53.48**	**40.57**	**53.90**	**48.48**	46.44	**59.09**	**46.10**

In the first ablation experiment, DRI-MVSNet used only the CVSF and MDRP to evaluate the importance of the CSCP to the network. The results in Table 4 show that the accuracy of DRI-MVSNet when it used the CSCP was 0.004 higher than that when it was not used. This is because the CSCP allows us to fully consider contextual information in the channel and the spatial dimensions of the pixel-related features. The introduction of the CSCP enhanced the feature representation capability of the proposed method. Table 5 shows that the F1-score increased by 4.65 when the CSCP was used. Its role was more prominent on the Tanks & Temples dataset because this is a large-scale dataset of outdoor scenes, and the CSCP can learn more information on spatial interdependence on such datasets, where this is important for pixel matching.

In the second ablation experiment, we did not use the CVSF. DRI-MVSNet was thus reduced to a network architecture that did not consider the similarity of pixel pairs in different views. As is shown in Table 4, the DRI-MVSNet with the CVSF was better by 0.003 and 0.011 than without it in terms of accuracy and completeness, respectively. In terms of overall values, the performance of DRI-MVSNet when using the CVSF was 0.008 better than that without it. Table 5 shows that the F1-score increased by 3.59 because the CVSF learns the similarity map under different views and at different depths, which makes it effective for reconstructing outdoor objects with a long-distance depth range.

The third ablation experiment was used to verify the effectiveness of the MDRP. In this experiment, we used only the CSCP and CVSF, and compared the DRI-MVSNet when using the MDRP with that without the MDRP. As shown in Table 4, the accuracy of the network with MDRP improved from 0.549 to 0.432 and its completeness improved from 0.415 to 0.327. As shown in Table 5, the F1-score increased by 9.93 using the MDRP was used than that without it. The experiment also proved the effectiveness of the non-uniform depth sampling strategy in the MDRP.

### Run-time and GPU memory

Table 6 shows the comparison between the other methods and DRI-MVSNet in terms of runtime and GPU memory. DRI-MVSNet consumed a minimal amount of memory and had low time complexity. Compared with Point-MVSNet-HiRes, its memory consumption and time complexity were lower by 5.5 GB and 4.52 s, respectively, and its overall performance decreased by only 0.003 because large-scale input images and depth maps needed to be processed.

**Table 6:** Comparisons of time complexity and memory consumption on the DTU dataset.

Methods	Input Size	Depth number	Depth Map Size	GPU Memory	Runtime	Overall
MVSNet [39]	1600x1184	256	400x296	15.4GB	1.18s	0.462
R-MVSNet [48]	1600x1184	512	400x296	6.7GB	2.35s	0.417
Point-MVSNet-HiRes [49]	1600x1152	96	800x576	8.9GB	5.44s	**0.376**
VA-Point-MVSNet [50]	1280x960	96	640x480	8.7GB	3.35s	0.391
DRI-MVSNet (Ours)	800x576	64,32,8	800x576	**3.4GB**	**0.92s**	0.379

## Discussion

The proposed DRI-MVSNet considers contextual information on the feature, similarity among pixels, and hypothetical depth planes for 3D reconstruction.

In this study, the CSCP module was proposed to fully capture channel-related and spatial information to improve the representation capability of each pixel feature. The experimental results show that this module improves the completeness and accuracy of the reconstructed point clouds.

The cross-view similarity-based feature map fusion module was proposed to learn the similarity between pixel pairs in the reference image and each source image at different depths. The cost volume constructed in this way better reflected the overall degree of matching of the pixels, and thus improved accuracy and completeness. As shown in Tables 2 and 3, our method delivered excellent results on the DTU and the Tanks & Temples datasets.

The multi-stage depth residual prediction module could accurately predict the depth residual prediction by using a non-uniform depth sampling strategy to construct the hypothetical depth planes. The non-uniform depth sampling strategy rendered the sampling of the hypothetical planes more reasonable and improved accuracy by 0.117.

## Conclusion

The large-scale 3D reconstruction of scenes remains challenging because details of features of the pixels are often ignored, and the task requires a large amount of memory. This study proposed a cascaded network for depth residual inference called DRI-MVSNet. This study makes three contributions to work in the area. First, the CSCP module was designed to capture contextual information between the features of pixels. Second, the cross-view similarity-based feature map fusion module, which was used to learn the degree of matching of pixel pairs between the source image and the reference image at different depths, was proposed. Finally, to iteratively refine the depth map, a multi-stage depth residual prediction module was proposed.

The proposed DRI-MVSNet delivered better performance on the DTU and the Tanks & Temples datasets than state-of-the-art methods, and generated a more accurate and complete 3D point cloud model. Moreover, extensive experiments proved its superior performance in terms of dense 3D reconstruction. However, DRI-MVSNet does not consider the impact of unpredictable noise in the scene. In future work, we plan to improve our network in terms of filtering noise to improve its accuracy of reconstruction.

## Supporting information

S1 Table Detailed performance comparison on DTU data set. (XLSX)

S2 Table Detailed performance comparison on T&T data set. (XLSX)
